# In vivo assessment of the host reactions to the biodegradation of the two novel magnesium alloys ZEK100 and AX30 in an animal model

**DOI:** 10.1186/1475-925X-11-14

**Published:** 2012-03-20

**Authors:** Tim Andreas Huehnerschulte, Janin Reifenrath, Brigitte von Rechenberg, Dina Dziuba, Jan Marten Seitz, Dirk Bormann, Henning Windhagen, Andrea Meyer-Lindenberg

**Affiliations:** 1 School of Veterinary Medicine Hanover, Small Animals Clinic, CRC 599, Bünteweg 9, 30559 Hanover, Germany; 2 University of Zurich, Muscoskeletal Research Unit, Winterthurerstrasse 260, 8057 Zurich, Switzerland; 3 Leibniz University of Hanover, Institute of Materials Science, An der Universität 2, 30823 Garbsen, Germany; 4 Medical School Hanover, Annastift, Anna-von-Borries-Straße 1-7 30625 Hanover-Kleefeld, Germany; 5 Faculty of Veterinary Medicine, Ludwig-Maximilians-Universitaet Muenchen, Veterinärstraße 13, 80539 Munich, Germany

**Keywords:** Magnesium, In vivo, Biocompatibility, Degradation, μ-computed tomography, Histology

## Abstract

**Background:**

Most studies on biodegradable magnesium implants published recently use magnesium-calcium-alloys or magnesium-aluminum-rare earth-alloys.

However, since rare earths are a mixture of elements and their toxicity is unclear, a reduced content of rare earths is favorable. The present study assesses the in vivo biocompatibility of two new magnesium alloys which have a reduced content (ZEK100) or contain no rare earths at all (AX30).

**Methods:**

24 rabbits were randomized into 4 groups (AX30 or ZEK100, 3 or 6 months, respectively) and cylindrical pins were inserted in their tibiae. To assess the biodegradation μCT scans and histological examinations were performed.

**Results:**

The μCT scans showed that until month three ZEK100 degrades faster than AX30, but this difference is leveled out after 6 months. Histology revealed that both materials induce adverse host reactions and high numbers of osteoclasts in the recipient bone. The mineral apposition rates of both materials groups were high.

**Conclusions:**

Both alloys display favorable degradation characteristics, but they induce adverse host reactions, namely an osteoclast-driven resorption of bone and a subsequent periosteal formation of new bone. Therefore, the biocompatibility of ZEK100 and AX30 is questionable and further studies, which should focus on the interactions on cellular level, are needed.

## Background

Recently, magnesium alloys returned to the focus of research as potential material for degradable metallic implants [1-10]. Besides problems like rapid corrosion, accumulation of subcutaneous gas and insufficient mechanical stability, adverse host reactions and toxic effects had also been limiting factors of the magnesium implants used by first researchers [11-15] and were reasons why magnesium had been abandoned. In modern magnesium alloys ligands are used to modify the corrosion properties and the mechanical characteristics of the alloy [5,6,16,17] and that is why modern magnesium alloys have favorable mechanical characteristics [1,3]. The magnesium alloys most commonly researched on are magnesium-calcium-alloys and magnesium-aluminum-rare earth-alloys [3,18-21]. They were shown to be of good in vivo biocompatibility [1,3,22]. Recent studies proved that they have no allergenic or sensitizing potential [23,24]. Furthermore, it has been published that magnesium has osteoinductive effects [1,3,7,25,26].

Although for some of the rare earth elements (RE) used as alloying components of magnesium unwanted effects have been reported, their toxicity is still widely unknown [27-29]. Thus, despite favorable mechanical properties, a magnesium alloy can only be considered suitable, if the released elements during the degradation of a magnesium implant are of acceptable biocompatibility [30,31]. Although good biocompatibility is mandatory for future uses [24], according to a recent study most studies on degradable magnesium alloys focus on material science and engineering aspects [30].

An assessment of the in vivo reactions, such as foreign body or immunologic reactions should be done in the recipient tissue [32]. Since rare earths are possibly toxic, a reduced content in the alloys might be favorable. ZEK100 and AX30 are two novel magnesium alloys, that have a reduced content of RE (ZEK100) or contain no rare earths at all (AX30) and which were shown to be *in vitro *promising [33].

The present study is a primary assessment of the in vivo host reactions to the two novel magnesium alloys ZEK100 and AX30 in the same in vivo setup as the preliminary studies [8,9].

## Methods

### Implant material

The two magnesium alloys used in this study were especially designed and made by the Institute of Material Science, University of Hanover, Germany.

ZEK100 consists of magnesium with 1 wt% of zinc, less than 1 wt% of zirconium as well as less than 1 wt% of rare earths and AX30 consists of magnesium with 3 wt% of aluminum and less than 1 wt% of calcium. They were both named in accordance with the ASTM standard B275-90 [34].

The ZEK100 and AX30 billets were manufactured by gravity die-casting. Due to the high reactiveness of liquid magnesium, it was melted and cast under a protective argon atmosphere, which was achieved by dynamically circulating argon around the crucible at a volumetric flow rate of 3 l/min. Both alloys were melted at a temperature of 760°C. The die used for the casting was heated to 600°C for ZEK100 and to 560°C for AX30. The billets were further processed by direct extrusion. For this purpose, their diameter was reduced to 120 mm by turning on a lathe. Then the billets were soaked at 350°C in a furnace for two hours, while the extrusion die (orifice diameter of 30 mm) and its recipient were heated to a temperature of 380°C for ZEK100 and to 400°C for AX30. Afterwards the billet was extruded at a ram speed of 1 mm/s for ZEK100 and 1.5 mm/s for AX30. The final implants were 2.5 mm in diameter and 25 mm in length.

All implants were washed in acetone and distilled water in an ultrasonic bath and then separately packed. They were sterilized with gamma radiation at 25 kGy for 8 h by a commercial provider (BBF Sterilisationsservice, Kernen, Germany) [1,3].

### Animal model

The animal experiments carried out in this study were in accordance with a protocol approved by the ethic committee in charge as well as with § 8 of the German Animal Welfare Act. They were legitimized by the Office for Consumer Protection and Food Safety under the approval number 33.9-42502-04-07/1363.

For the experiment 24 female, adult New Zealand White Rabbits (Charles River, Kisslegg, Germany) with a body weight of 3.5 to 4.5 kg were used. The rabbits were housed in separate cages in a controlled environment.

The animals were randomized into four groups of six animals each differing in time and/or material (AX30 3 months, AX30 6 months, ZEK100 3 months and ZEK100 6 months). The implants were placed in the right tibiae of the animals. Within each group there was one animal without an implant, which served as negative control, resulting in 20 implants in total and two negative controls for three months as well as two for six months.

Before the operation procedure all rabbits received subcutaneous injections of meloxicam (0.15 mg kg^-1^, Metacam^®^, Boehringer Ingelheim, Ingelheim, Germany) and enrofloxacin (10 mg kg^-1^, Baytril^® ^2.5%, Bayer HealthCare, Leverkusen, Germany). This medication was continued orally during the following ten days post operatively. To induce anaesthesia, the rabbits received intramuscular injections of s-ketaminehydrochloride (10 mg kg^-1^, CP-Pharma, Burgdorf, Germany) and medetomidine (0.125 mg kg^-1^, Domitor^®^, Pfizer GmbH, Berlin, Germany). After endotracheal intubation, the anaesthesia was continued by administering a mixture of isoflurane and oxygen (2 to 3 vol% isoflurane, oxygen airflow 0.4 to 0.6 l min^-1^, Isoba^®^, Essex Pharma GmbH, Munich, Germany) under spontaneous respiration. Furthermore, the rabbits received an infusion of Paediafusin^© ^(10 ml kg^-1 ^h^-1^, Baxter, Unterschleissheim, Germany). The right hind legs were clipped and the rabbits were placed on a heating pad. Shortly before the incision fentanyldihydrogencitrate (10 μg kg^-1^, Fentanyl-Janssen^®^, Janssen-Cilag GmbH, Neuss, Germany) was given intravenously and from that time the rabbits were ventilated artificially if necessary.

An incision of the skin and the fascia underneath was made on the medial side of the tibia, just mediodistal of the tibial tuberosity. After the periosteum had been detached from the tibia, a 2.5 mm wide hole was drilled through the cortex, so that the implant could be placed in the middle third of the medullary cavity using a sterile plastic push. The soft tissue layers were closed separately. After the operation the position of the implants was confirmed radiographically in two projections.

In the control animals the operation procedure was performed as described above, including the insertion of the push, except for no implant was inserted.

During the follow up the animals were examined clinically on a daily basis. Special attention was paid to the occurrence of pain, lameness, subcutaneous accumulation of gas or swellings. Four fluorochromes (Calcein green, Xylenol orange, Calcein blue and Tetracycline) were administered subcutaneously according to a protocol shown in Table 1.

**Table 1:** Scheme of Fluorochrome staining

Fluorochrome	Dose [a]	3 month groups [b]	6 month groups [b]
Calcein green (1%)	1	3 and 6	93 and 96
Xylenol orange (10%)	1	33 and 36	120 and 123
Calcein blue (2%)	1.5	60 and 63	150 and 153
Tetracycline (10%)	0.3	89 and 92	179 and 182

[a] Dose in ml per kg bodyweight [b] Numbers indicate the day postoperatively at which the respective Fluorochrome was administered

After three or six months the animals were anaesthetized and then euthanized by intracardiaic injections of narcobarbital (230 mg kg^-1^) and their right tibiae were explanted and fixated in buffered 4% formaldehyde.

### Postmortem micro computed tomography (μCT) scans

Scans of the isolated tibiae were performed using a cone beam μCT scanner (μCT80, Scanco Medical, Zurich, Switzerland), with a maximum resolution of 5 μm and maximum image matrix of 4096 × 4096 pixels. The scanner uses a 2D and true 3D multi-planar reformatting evaluation and visualization software (Scanco Medical, Zurich, Switzerland), which allows volume registration and 2D and 3D density measurements of user defined regions of interest. 3D analysis scripts allow further processing of irregularly shaped three-dimensional volumes of interest (VOI).

For the scans the tibiae were fixated in cylindrical tubes filled with 4% formaldehyde using small sponges. The tubes could be placed in the μCT scanner. Topograms of the legs were made and then a scan area from 5 mm above to 5 mm below the implant was determined for the tomogram. The slice thickness was 36 μm and the integration time used was 1 sec per slice. The electron energy used was set to be 55 kVp and the intensity was 72 μA.

The parameters to be analyzed were: volume, density and 3D thickness of the implant and the 3D thickness variation. For evaluation the implant was manually contoured in the 2D images. A threshold, specific for the two different alloys (ZEK100 threshold 204 and AX30 threshold 185), was determined and used for all evaluations. This procedure defined a three-dimensional VOI, which could be further evaluated. In order to assess the extents of the degradation of the implants, volumetric and density measurements of the VOI were performed. The average density of the VOI was measured and stated in arbitrary units (AU), because the μCT was calibrated using a hydroxyapatite phantom for bone density measurements. The direct 3D determinations of thickness of the VOIs were calculated by filling the structure with overlapping spheres of maximal diameter. The diameter of the spheres at each location resembles the local thickness and the average thickness was determined by averaging them over the whole VOI.

This led to histograms of bin sizes with an average 3D thickness and a standard deviation for each implant. A low average bin size with a low standard deviation indicates a high degree of uniform corrosion. A high standard deviation of the histogram is caused by an irregular shape of the remaining implant and therefore it is an indicator for the extent of pitting corrosion. Additionally to the volumetric method described above, per animal nine images, evenly distributed over the 2D images of the topograms, were made. They were used for the measurements of the cross sectional areas of the implants, which were done with the AxioVision Release 4.8.2 (Carl Zeiss AG, Jena, Germany) in accordance to a protocol published previously [9,35], by manually contouring the implant in the 2D images with the area measurement tool of the software.

Furthermore, to assess bone changes as a measure for the biocompatibility, the nine 2D images obtained of the topograms were scored using a semiquantitative scoring system (Table 2), modified after one used in a previous study [9]. The scores of each animal were summed up to a total score. The scoring allocated values between 0 and 3 for defined features in the 2D images of the μCT scans.

**Table 2:** Score for the 2D images in the μCT80

Feature		Score
Bone structure (cavities)	regular	0
	minor irregularities (< 30% of the area)	1
	distinct irregularities (30 to 60% of the area)	2
	severe irregularities (< 60% of the area)	3
Bone implant contact (trabeculae)	none	0
	< 1/3 of the implant surface	1
	1/3 to 2/3 of the implant surface	2
	> 2/3 of the implant surface	3
Endosteal formation of new bone	none	0
	< 1/3 of the endosteal surface	1
	1/3 to 2/3 of the endosteal surface	2
	> 2/3 of the endosteal surface	3
Periosteal formation of new bone	none	0
	< 1/3 of the periosteal surface	1
	and < 1/3 of the cortical thickness	
	1/3 to 2/3 of the periosteal surface	2
	or 1/3 to 2/3 of the cortical thickness	
	> 2/3 of the periosteal surface or > 2/3 of the cortical thickness	3

### Histological preparation and analysis

After the fixation of the bone-implant-complex in formaldehyde and the examinations in the μCT80, the tibia was embedded in methyl-methacrylate (Technovit 7200 VLC, Heraeus Kulzer GmbH, Wehrheim, Germany) in accordance with the instructions of the manufacturer. Slices were produced using the sawing and grinding technique described by Donath [36].

Slices that were to be stained with Toluidine blue (n = 48) or Tartrate-resistant acid phosphatase (TRAP) (n = 24) were cut to a thickness of about 100 μm and then ground and polished to a final thickness of approximately 50 μm. Additionally one native slice per animal, with a final thickness of 100 μm, was subject to histomorphometrical analyses of in vivo fluorochrome labels.

**Figure 1:**
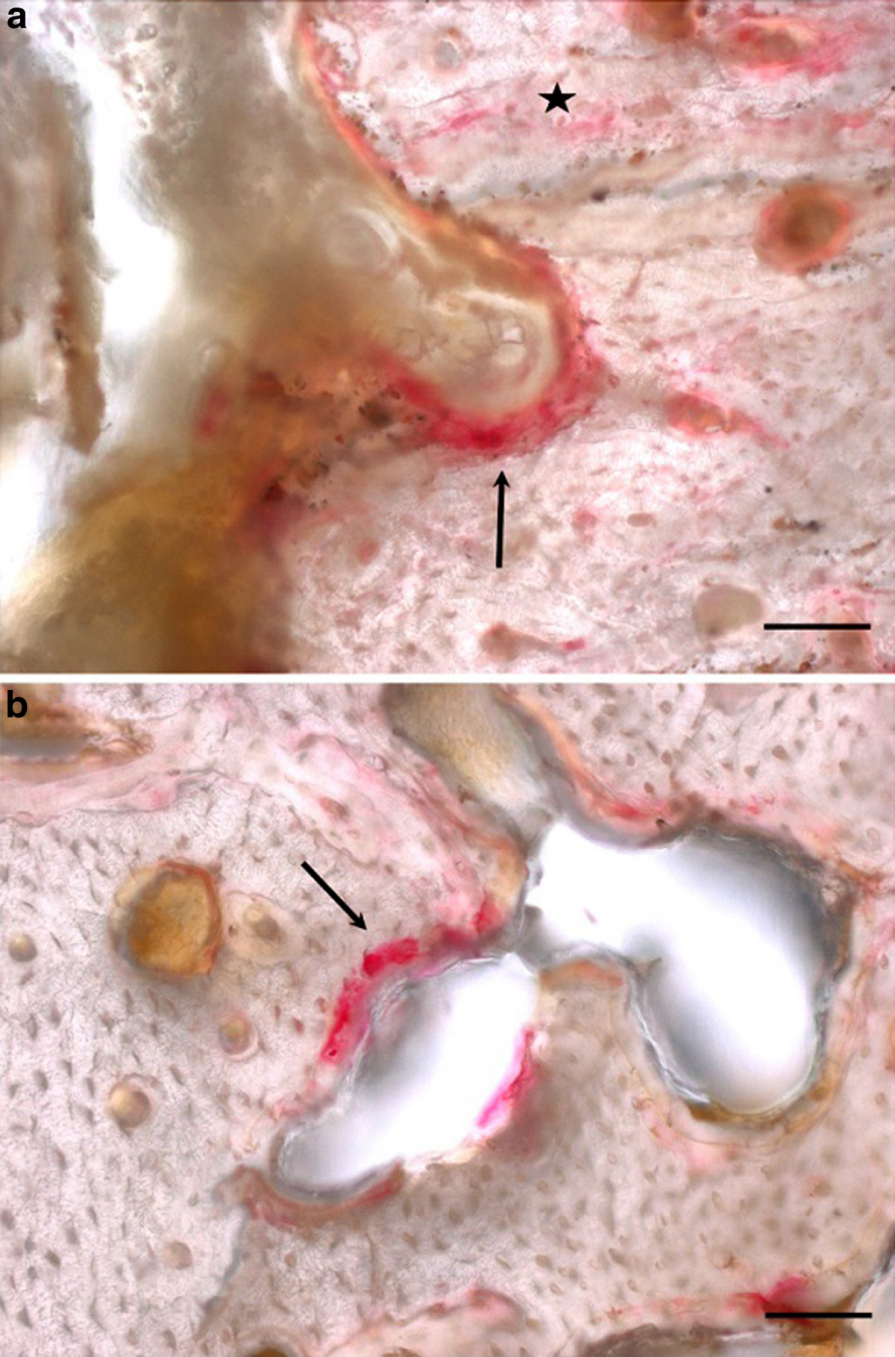
**Details of osteoclasts in TRAP staining**. [a] Osteoclast on endosteal surface [b] Cortical cavity with osteoclasts. Black arrows: TRAP positive bone of Howship-lacunes, black star: TRAP positive staining of cement lines, black bar: scale bar 50 μm.

For the TRAP staining the slices were etched in 1% acetic acid and then rinsed with deionised water. After that they were bathed in 0.2 M acetate buffer for 45 min. The final staining was done by immersion in TRAP staining solution (naphthol AS-MX phosphate and Fast Red TR Salt, Sigma-Aldrich, St. Louis, USA) in 80 ml 0.2 M acetate buffer) for 90 min at 37°C. After that the slices were again rinsed with deionised water. Osteoclasts were stained in bright red while the rest of the slice remained unstained [37]. In the stained slices the osteoclasts were counted by an indirect method. Since the osteoclasts did not stain in the thick undecalcified slices and often were detached from the osseous surfaces, instead of the osteoclasts themselves, the intensely reddish stained bone areas in the Howship-lacunas (Figure 1) were counted. This indirect osteoclast counting was done three times for each slice and averaged. For the Toluidine blue staining the slices were etched in 0.7% formic acid for 4 min, then patted dry and stained for 15 min at 60°C in the Toluidine blue staining solution (0.1% Toluidine blue O (Chroma, Muenster, Germany) solution in the phosphate buffer) and then rinsed with deionised water and dried at ambient air. Toluidine blue stained slices are coloured in shades of blue. The mature bone appeared in light blue, while new bone was of dark blue [38].

**Figure 2:**
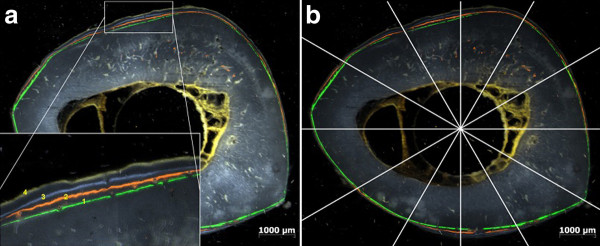
**Fluorochrome labeling for histomorphometry**. [a] Labels of the four fluorochromes used (1 Calcein green, 2 Xylenol orange, 3 Calcein blue, 4 Tetracycline) [b] Positions for the measurement of the distance between the labels (same slices)

All histological analyses were done using a Zeiss AxioImager Z1 and the AxioVision software release 4.8.2. (Carl Zeiss AG, Jena, Germany). For the Toluidine blue stained slices a semiquantitative scoring was used to quantify the bone features observed in the slices (Table 3). A total score for the bone reactions was calculated by adding up the different scores given for each animal. The histomorphometrical measurements were done with native slices on the basis of previous studies on fluorochrome labeling, but modified to the needs of the present study [39-41]. Distances between the double labels of the successionally administered fluorochromes were measured in the periosteally formed bone at twelve defined locations (Figure 2). For each animal the mineral apposition rates (MAR) [42] of the respective time spans were calculated as average of the twelve locations.

**Table 3:** Semiquantitative scoring of Toluidine blue stained slices

Feature		Score
Assessment of the bone structure (cavities)	regular	0
	minor irregularities (< 30% of surface)	1
	distinct irregularities (30 to 60% of surface)	2
	severe irregularities (< 60% of surface)	3
Periosteal remodelling	none	0
	thickness equals 1 osteon	1
	thickness equals 2 osteons	2
	thickness equals 3 osteons	3
Endosteal remodelling	none	0
	thickness equals 1 osteon	1
	thickness equals 2 osteons	2
	thickness equals 3 osteons	3
Endosteal formation of new bone	none	0
	< 1/3 of the endosteal surface	1
	1/3 to 2/3 of the endosteal surface	2
	> 2/3 of the endosteal surface	3
Periosteal formation of new bone	none	0
	< 1/3 of the periosteal surface	1
	and < 1/3 of the cortical thickness	
	1/3 to 2/3 of the periosteal surface	2
	or 1/3 to 2/3 of the cortical thickness	
	> 2/3 of the periosteal surface	3
	or > 2/3 of the cortical thickness	

### Statistics

All statistical tests were done with the programs Microsoft Office Excel^®^, Version 2003 (Microsoft Cooperation, Redmond, USA) and SPSS^® ^Version 17.0 (SPSS: An IBM Company, Chicago, USA).

For statistical analyses all results were checked for normal distribution. The results of the direct 3D evaluations and the area measurements of the implants in the μCT turned out to be normally distributed and therefore Student's t-tests were used for comparisons between materials and time points. For the numbers of osteoclasts in the TRAP-stained slices and the MAR mean values and standard deviations were calculated and an ANOVA analysis with subsequent post hoc tests (Games-Howell) was performed. The scores of the 2D μCT images and the Toluidine blue stained slices were averaged for each animal and then the minimum, median and maximum of each group was determined. For each time point Kruskal-Wallis-tests with subsequent Mann-Whitney-U-tests were done. The level of significance for all statistical analyses was *p *≤ 0.05.

## Results

In the clinical examinations performed during the follow up period in all animals minor swellings and mild wound reactions surrounding the incision could be found for up to the first ten days. No lamenesses were seen.

The results of the direct 3D measurements of the implants are displayed in Table 4 and the corresponding p-values are shown in Table 5. The volume and the direct 3D thickness of both materials decreased time dependently, although not significantly. The standard deviation of the 3D thickness was higher in the 6 months groups of both materials than in the respective 3 months groups, but only for AX30 this difference was significant. The implants of the AX30 3 months group had a significantly higher volume and 3D thickness as well as a significantly lower density and 3D variation than the ZEK100 3 months implants. After six months the volume and 3D thickness of the AX30 6 months group was still higher than that of the ZEK100 group and the 3D variation lower than that of the ZEK100, though not significantly. Significant differences in implant density could only be found between AX30 and ZEK100 after six months.

**Table 4:** Results of the 3D measurements of the μCT scans

Group	n	Volume		Density		3D thickness		3D variation	
		[mm^3^]		[1/cm]		[mm]		[mm]	
		MV	SD	MV	SD	MV	SD	MV	SD
AX30 3 months	5	116.52	1.36	2.14	0.08	2.25	0.06	0.25	0.06
AX30 6 months	4	102.34	14.62	2.23	0.19	1.87	0.42	0.38	0.10
ZEK100 3 months	5	105.90	10.62	2.62	0.12	1.99	0.21	0.33	0.07
ZEK100 6 months	3	99.22	5.69	2.57	0.62	1.82	0.15	0.43	0.60

**Table 5:** p-values of the 3D measurements in the μCT scans

Material	Time point	Volume	Density	3D thickness	3D variation
AX30	3 vs. 6 months				*p *= 0.043
ZEK100	3 vs. 6 months				
AX30 vs. ZEK100	3 months	*p *< 0.001	*p *< 0.001	*p *= 0.001	*p *= 0.011
AX30 vs. ZEK100	6 months		*p *= 0.007		

The average cross sectional areas are displayed in Table 6. Since there were no initial μCT investigations of the implants, origin cross sectional areas were calculated to be 4.91 mm^2^. Compared to this the implants of both materials had a markedly reduced diameter after three months. However, statistical analyses between calculated initial values and measured values after three and six months were not performed due to different evaluation methods. The implants of the AX30 6 months group had a significantly lower (*p *= 0.019) cross sectional area than those of the AX30 3 months group, while for the ZEK100 6 months group it was lower than that of the ZEK100 3 months group, but not significantly. The average cross sectional area of the ZEK100 3 months group was significantly lower than that of the AX30 3 months group (*p *= 0.014). But after six months this difference was equalized and both materials had about the same average cross sectional areas.

**Table 6:** Results of the cross sectional area measurements

Group	n	MV [mm^2^]	SD [mm^2^]
AX30 3 months	5	4.73	0.07
AX30 6 months	5	3.95	0.56
ZEK100 3 months	5	4.13	0.44
ZEk100 6 months	5	3.91	0.34

The results of the scoring of the 2D μCT images are presented in Table 7. There were no statistically significant differences between the two materials and the control group except in the parameter endosteal formation of new bone after three months (*p *= 0.032), although the median scores of the material groups were markedly higher than those of the respective control groups. For the two time groups of each material there were also no significant differences. In contrast to the control, the magnesium implant groups were found to have diffusely distributed cavities in the cortical bone, which appeared to be located rather close to the endost than the periost. Although in both materials there were more cavities after six months than after three months the difference was not significant due to higher standard deviations.

**Table 7:** Resulting scores of the 2D μCT images

Group	n		Overall bone structure (cavities)	Bone implant contact (trabaeculae)	Endosteal formation of new bone	Periosteal formation of new bone	Total score
AX30 3 months	5	Min	0.22	0.00	0.00		0.22
		**Med**	**1.22**	**0.00**	**0.11**	**1.67**	**3.00**
		Max	2.89	0.11	0.44		3.44
AX30 6 months	5	Min	0.11	0.00	0.00	1.00	1.11
		**Med**	**2.11**	**0.00**	**0.22**	**2.44**	**4.77**
		Max	3.00	0.44	0.67	3.00	7.11
ZEK100 3 months	5	Min	0.00	0.00	0.00	0.00	0.00
		**Med**	**1.11**	**0.00**	**0.00**	**2.67**	**3.78**
		Max	2.67	0.00	0.00	3.00	5.67
ZEK100 6 months	5	Min	1.00	0.00	0.00	1.00	2.00
		**Med**	**2.67**	**0.00**	**0.00**	**2.22**	**4.89**
		Max	3.00	0.00	0.00	3.00	6.00
Control 3 months	2	Min	0.00	-	0.00	0.00	0.00
		**Med**	**0.00**	**-**	**0.00**	**0.00**	**0.00**
		Max	0.00	-	0.00	0.00	0.00
Control 6 months	2	Min	0.00	-	0.00	0.00	0.00
		**Med**	**0.00**	**-**	**0.00**	**0.00**	**0.00**
		Max	0.00	-	0.00	0.00	0.00

**Figure 3:**
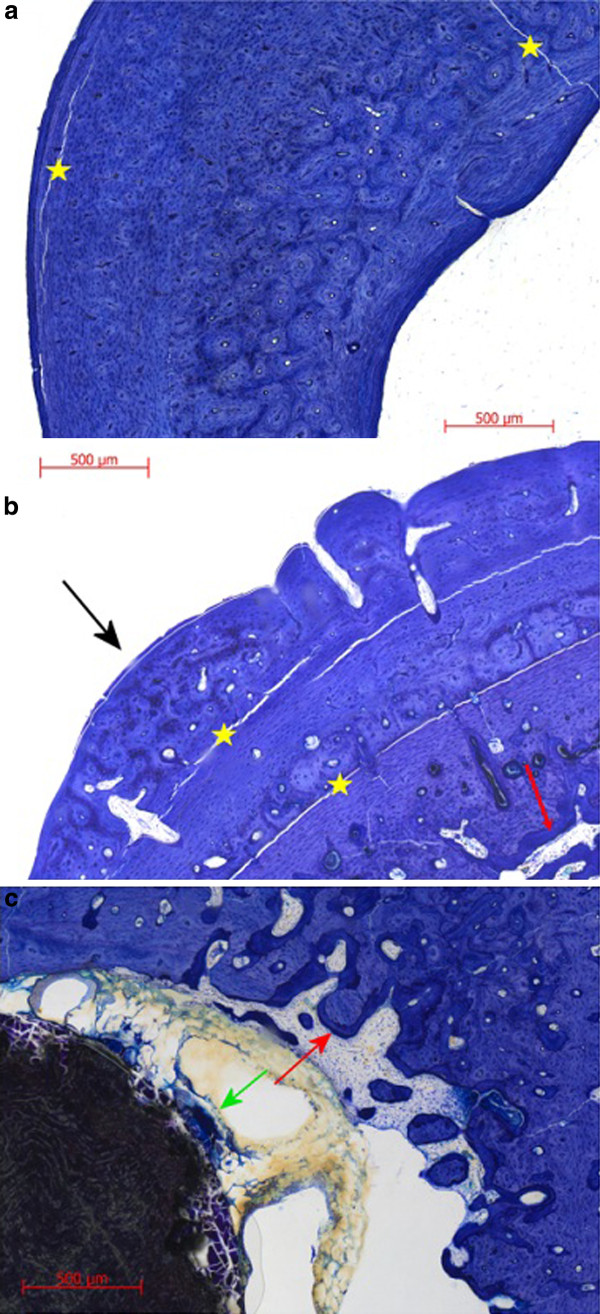
**Details of Toluidine blue stained slices**. [a] slice of an animal of the 6 months control group. [b] slice of the ZEK100 6 months group. [c] slice of the ZEK100 6 months group (yellow star: artefact due to preparation, white arrow: periosteal remodelling, red arrow: endosteal remodelling, black arrow: periosteal formation of new bone, green arrow: periimplant fibrosis)

The results of the scoring of the Toluidine blue stained slices are displayed in Table 8 and examples of the features that were scored are shown in Figure 3. The *p*-values of the differences between the respective scores are displayed in Table 9. After three as well as six months the ZEK100 and the AX30 implants (total score 8.5 to 11.5) induced a higher degree of host reactions than in the control groups (total score 1.5 to 2). This difference was significant for the total score and most of the single parameters. The only significant difference between the different parameters of the respective implant groups was the periimplant fibrosis, which could not be seen in the AX30 3 months group. The parameters bone structure (cavities), periosteal remodelling and periosteal formation of new bone had high score values while the parameters bone implant contact, endosteal remodelling and endosteal formation of new bone had lower values.

**Table 8:** Scores of the Toluidine blue stained slices

Group	N		Bone structure (cavities)	Periosteal remodelling	endosteal remodelling	Endosteal formation of new bone	Periosteal formation of new bone	Bone implant contact (trabeculae)	Periimplant fibrosis	Total score
**AX30 3 months**		Min	0	2	0	0	3	0	0	6
	**10**	**Med**	**1**	**3**	**1**	**0**	**3**	**0**	**0**	**8.5**
		Max	3	3	2	0	3	0	0	10
**AX30 6 months**		Min	0	1	0	0	2	0	1	7
	**10**	**Med**	**2.5**	**3**	**1**	**0**	**3**	**0**	**3**	**11.5**
		Max	3	3	1	2	3	0	3	15
**ZEK100 3 months**		Min	1	2	0	0	3	0	0	6
	**10**	**Med**	**2**	**3**	**1**	**1**	**3**	**0**	**3**	**11**
		Max	3	3	1	2	3	0	3	14
**ZEK100 6 months**		Min	1	1	0	0	0	0	2	6
	**10**	**Med**	**1**	**3**	**1**	**0.5**	**3**	**0**	**3**	**10**
		Max	3	3	3	1	3	0	3	14
**3 months control**		Min	0	0	0	0	0			1
	**4**	**Med**	**0**	**1**	**1**	**0**	**0**	**-**	**-**	**1.5**
		Max	0	1	1	0	0			2
**6 months control**		Min	0	0	0	0	0			**0**
	**4**	**Med**	**0**	**1**	**1**	**0**	**0**	**-**	**-**	**2**
		Max	0	1	1	0	0			**2**

**Table 9:** *p*-values for the results of the Toluidine blue stained histological slices

Group [a]	Time point	Assessment of bone structure (cavities)	Periosteal remodelling	Endosteal formation of new bone	Periosteal formation of new bone	Periimplant fibrosis	Total score
AX30 and ZEK100	3	-	-	-	-	0.002	0.003
AX30 and control	3	0.008	0.002	-	0.002	-	0.002
ZEK100 and control	3	0.002	0.003	-	0.003	0.002	0.002
AX30 and ZEK100	6	-	-	-	-	-	-
AX30 and control	6	0.002	0.002	-	0.002	0.024	0.002
ZEK100 and control	6	0.024	0.024	-	0.008	0.004	0.002
AX30	3 and 6	-	-	-	-	< 0.001	-
ZEK100	3 and 6	-	-	-	-	-	-

[a] p-values at respective time points in the Mann-Whitney tests of the parameters with significant differences in the Kruskal-Wallis test

**Figure 4:**
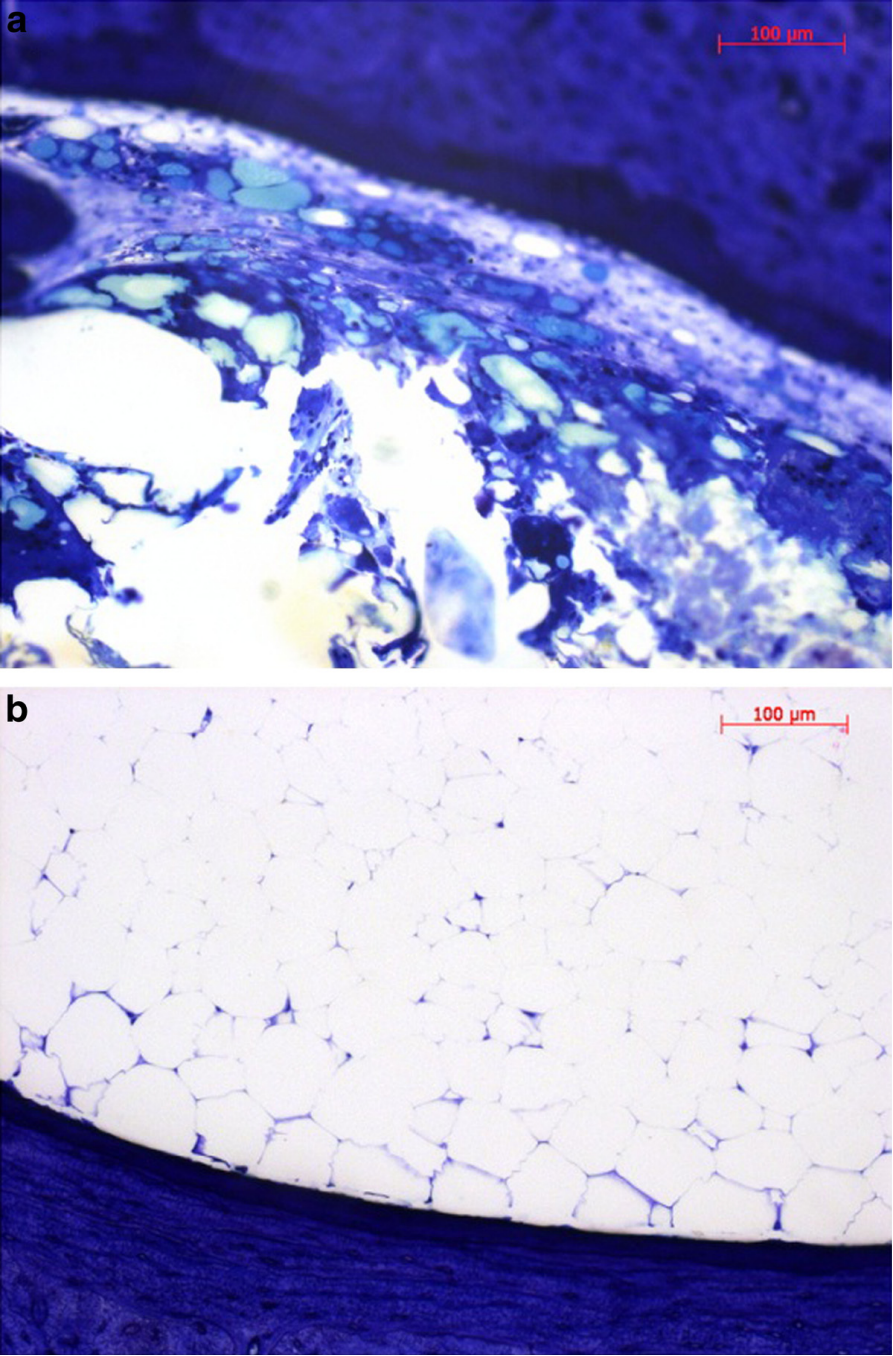
**Details of cortical bone and adjacent bone marrow**. [a] 3 months control group [b] ZEK100 3 months group.

**Figure 5:**
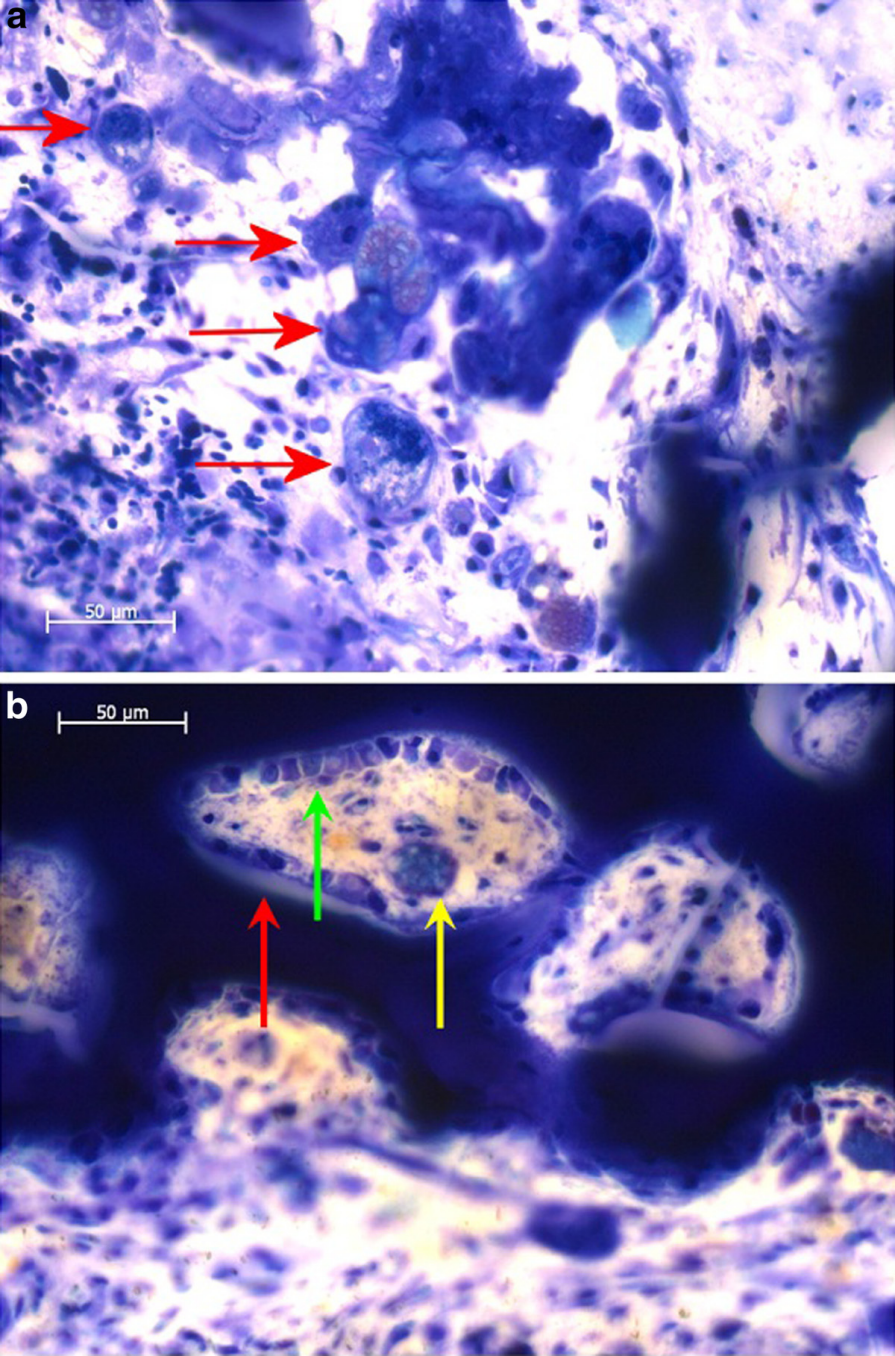
**Details of a Toluidine blue stained slice of the ZEK100 3 months group**. [a] Bone marrow between endost and implant (red arrows: foreign body giant cells) [b] Bone marrow close to endost with trabecular formation of new bone (green arrows: osteoneogenesis, layer of osteoblasts on light unmineralised osteoid and dark blue mineralised bone, red arrow: endosteal remodelling, yellow arrow: macrophage)

The histological preparation caused losses of material from the medullary cavities. Therefore a quantification of the cells found could not be done. Furthermore, the slice thickness of 50 μm made morphological evaluations difficult. In the control groups the medullary cavities were homogenously filled with a bone marrow of mainly fat-containing cells, while in the implant groups a cell-rich bone marrow was found that contained fibrous tissue as well as implant- and cell debris. (Figure 4) In areas of the bone marrow adjacent to the implant cells of an inflammatory reaction, such as macrophages and foreign body cells, were observed. (Figure 5) At sites of osteoneogenesis or remodelling linings of osteoblasts on layers of unmineralized osteoid were frequently found. (Figure 5)

In the TRAP staining it was differentiated whether osteoclasts were located endosteally or cortically (Figure 1). For both materials the TRAP staining revealed higher numbers of osteoclasts in the 3 months groups than in the 6 months groups (Table 10). In the control groups there were no or only very few osteoclast. No statistically significant differences existed between the implant and the control groups.

**Table 10:** Numbers of osteoclasts in TRAP staining

Group	n		Endosteal	Cortical	Total
**AX30 3 months**	5	**MV**	7.4	41.0	48.4
		**SD**	6.7	29.0	34.8
**AX30 6 months**	5	**MV**	7.5	14.7	22.2
		**SD**	6.3	10.4	15.4
**ZEK100 3 months**	5	**MV**	15.3	35.5	50.9
		**SD**	20.3	8.2	23.3
**ZEK100 6 months**	5	**MV**	7.1	25.9	33.0
		**SD**	8.4	22.7	27.2
**control 3 months**	2	**MV**	0.0	0.3	0.3
		**SD**	0.0	0.3	0.3
**control 6 months**	2	**MV**	0.0	0.0	0.0
		**SD**	0.0	0.0	0.0

**Figure 6:**
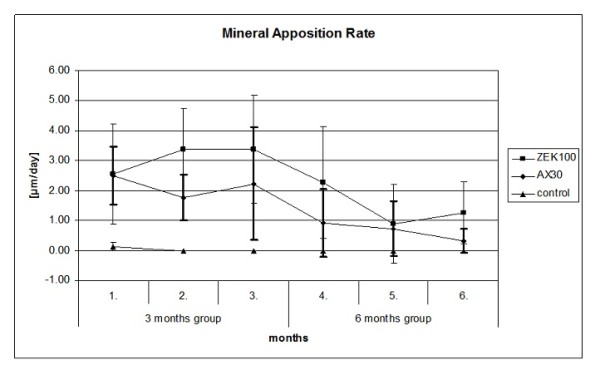
**Average periosteal mineral apposition rate (MAR)**.

The results of the histomorphometrical measurements are displayed in Table 11. Although the ZEK100 groups generally had a higher MAR, due to the high standard variations no significant differences between the two materials at the respective time spans were found (Figure 6). Both materials had a comparable average MAR during the first month postoperatively. After three months the MAR of both material groups reduced. In the control groups only a very moderate periosteal mineral apposition was found and there was only little cortical remodeling.

**Table 11:** Average periosteal mineral apposition rate (MAR)

MAR [μm/day]	n		3 months group			6 months group		
			1.	2.	3.	4.	5.	6.
**ZEK100**	5	**MV**	2.55	3.38	3.37	2.27	0.90	1.27
		**SD**	1.67	1.39	1.81	1.87	1.32	1.02
**AX30**	5	**MV**	2.50	1.77	2.23	0.92	0.73	0.33
		**SD**	0.96	0.76	1.88	1.13	0.92	0.40
**control groups**	2	**MV**	0.13	0.00	0.00	0.00	0.00	0.00
		**SD**	0.13	0.00	0.00	0.00	0.00	0.00

## Discussion

The present study was designed to assess the in vivo host reactions to the biodegradation of the two new magnesium alloys ZEK100 and AX30 in an animal experiment.

The authors of the present study chose this in vivo approach, with an intramedullar implant mimicking an intramedullar fixation device, for two reasons: First, it is known that the clinical tolerance and reaction of the bone to a magnesium implant are indicators for its biocompatibility [9,32] and that they depend on the receptor tissue, organ and species, as well as the size and shape of the implant [43]. Secondly, an adequate evaluation of the biocompatibility of degradable implants must take the extent of their degradation into account [7,43,44] and that the degradation rates also depend on the location [7,45,46].

In accordance with other studies examining the in vivo degradation of magnesium implants the clinical tolerance of the implants was good [8,9,17,43,47], but contrary to these clinical results, the μ computertomographical and histological examinations revealed severe osseous reactions to the degrading implant.

The basic methods to determine the extent of degradation, like weight or volume measurements, need the bone implant complex to be destroyed and make histological analyses impossible. In cross sectional area measurements of radiographic or histological 2D images the true implant volume has to be extrapolated and therefore they are considered to be not exact [48].

Therefore, in the present study a μCT scanner was used to determine the extent of degradation by direct 3D measurements of the implant. A μCT based method requires a user to define a VOI by contouring and determining thresholds. Since methodic errors may arise from this [49,50], the extent of degradation was also assessed by cross sectional area measurements in 2D images to verify the results and to allow comparisons to previous studies.

The direct 3D measurements of the implant volume revealed a time dependency of the degradation. It was shown, although only significantly after three months, that ZEK100 had degraded to a greater extent than AX30. After six months the implants of both 6 months groups had degraded to about the same extend. This relation was also found in the cross sectional area measurements. The results of the 3D thickness and 3D thickness variation did not only show that AX30 had a more uniform degradation than ZEK100, but they also supported the results of the volumetric measurements. Presumably the surface of the AX30 implants showed a better initial corrosion resistance than ZEK100, which could be due to possible differences in the distribution of the alloying elements within the implant.

The magnesium alloys LAE442, WE43 and MgCa0.8 were analyzed in the same experimental setup as ZEK100 and AX30, where ZEK100 implants showed a degradation behavior like the favorable LAE442. In the first three months AX30 showed a slower degradation than LAE442 and ZEK100 but after six months it was comparable [8].

As in a previous study, the host reactions were assessed by a scoring of 2D μCT images [9]. The scores for the overall assessment of the osseous structure at three and six months showed, that both alloys induced distinct time dependant host reactions, in the form of structural changes of the bone, while the control groups did not show any of such reactions. After six months both materials had induced about the same degree of host reaction, while after three months in the ZEK100 animals the degree of host reactions was higher than in the AX30 animals. Since, as discussed above, ZEK100 degraded faster than AX30 in the first three months, a relation of the host reaction and the extent of the degradation seems likely. The parameters for endosteal and periosteal formation of new bone showed the same tendency.

A six months study on MgCa0.8 with and without fluoride coating assessing 2D μCT images revealed bone formation endosteally and around the implant, which was assigned to a good biocompatibility [51]. Contrary to that, in the present study no bone implant contact was found and the score for the endosteal formation of new bone was low.

For examinations of the host reactions to magnesium implants histology is the method of choice [52] and a requirement for the biological evaluation of medical implants [53]. In the assessment of implants for bones the two aspects of the host reactions to be examined are morphological changes of the bone and cellular or inflammatory reactions [52].

Therefore, in the present study the morphological changes of the bone and the cellular reactions were assessed histologically and histomorphometrically. Additionally, osteoclasts were quantified in TRAP staining.

The results of the scoring of the Toluidine blue stained slices revealed host reactions and therefore they are contradictional to those of the 2D μCT scoring. Some of the studies on implants made of magnesium or its alloys reported about the absence of inflammatory reactions adjacent to the implant [13,14]. Von der Hoeh et al. [54] found the inflammatory reactions, namely foreign body giant cells, macrophages, lymphocytes and plasma cells and fibrous reactions near by the implant, to depend on the corrosion rate of MgCa0.8 cylinders in the cancellous bone, but no morphological changes of the bone [54]. Analogous to that, a recent study found similar moderate inflammatory and fibrous reactions in soft tissues adjacent to MgCa0.8 implants [43]. Contrary, a study examining Toluidine blue stained histological slices of rabbit tibiae with degrading magnesium hydroxide cylinders found no inflammation histologically and clinically [26]. Most studies published on magnesium implants report about beneficial osteoinductive effects of magnesium alloys [1,7,9,26,51,55]. In contrast to the studies published in the first half of the last century, in the recent years of magnesium research, there is only one study published that found severe adverse reactions to be induced by magnesium implants [56]. However, in some of the in vivo studies on the degradation of magnesium implants, there were synchrotron-, μCT- or histological images published, that show cortices with diffusely distributed cavities of uncommented origin [48,51,57].

In the present study the TRAP staining was used to quantify osteoclasts. The high numbers of osteoclasts in the tibiae with implants and the absence of osteoclasts in the control groups show that the degradation of the magnesium implants must have lead to an activation or chemotaxis of osteoclasts. Surprisingly the tests for significance between the numbers of osteoclast between the implant groups and the control groups were not significant, which might be due to the small size of the respective control group. The high numbers of osteoclasts in the implant group are a likely explanation for the formation of cavities in the cortices. Verbrugge et al. [12] described lyses of the bone in reaction to degrading magnesium. Janning et al. [26] also used the TRAP staining to quantify numbers of osteoclasts around cylinders made of magnesium hydroxide. They reported an initial inhibition of osteoclasts, which lasted for four weeks postoperatively, and a distinct activation of osteoclasts at six weeks after operation. Unfortunately, six weeks was the longest group in their study, so the further progression of osteoclast numbers remains unclear. In the present study, there were more osteoclasts found in the 3 months groups of both materials than in the 6 months groups, which might be a hint that the osteoclastic activity is time dependant.

The MAR measurements showed that already one month after the implantation a periosteal apposition takes place. It has its peak about two to three months postoperatively and gradually reduces until month six. Since all rabbits used in the study were adult and since the control group showed only a minimal mineral apposition, the increased MAR can be attributed to the implants. The mineral apposition in the first month postoperatively found in the control groups is likely to have been caused by the operation routine, but since it was so minor it can be neglected for the implant groups.

In other studies that assessed the MAR in bones with degrading magnesium implants also an enhanced MAR was found, in comparison to the control [1,26]. Janning et al. [26] discussed the MAR to be at its highest two weeks postoperatively and to decrease over the course of week four to six.

The appearance of comparable adverse reactions in all implants groups shows, that the rare earths, as an alloys component of 1 wt%, have no pronounced effect on the in vivo biocompatibility.

As a possible explanation for the activation of osteoclasts and the subsequent formation of cavities in the cortical bone it is hypothesized as follows. It is known that metal debris and ions released from conventional implants cause, when being phagocytised by macrophages, an increased osteoclastic activity, that is mediated by various inflammatory, macrophage derived mediators (interleukin 6 among others) [58-61]. Corrosion products from magnesium implants and their debris could have the same effect. Released Mg^2+ ^can possibly inhibit the osteoclastic activity, for it is likely to have the same effect as Ca^2+ ^has [62-64], which is a membrane receptor mediated inhibition of osteoclasts in a dose dependant manner [59,62,65]. But later on this inhibition is overridden by the effects of other mediators, such as interleukins or the RANK/RANKL system [62,66,67]. For IL6 a high osteoclastogenic potential was proven [68] and that it fully reverses the inhibitory effect of Ca^2+ ^on osteoclasts [62,63]. Furthermore, Ca^2+ ^was described to potently enhance the synthesis and secretion of IL6 [69].

Mechanical strain is believed to induce the formation of new bone, for it is sensed by osteocytes and stimulates them to release transmitters, which induce the formation of new bone periosteally [70,71]. Resorption cavities cause locally heightened strain levels in the remaining adjacent bone [70] and thereby stimulate the formation of new bone [72,73].

The exact mechanisms of interactions between degrading magnesium alloys especially on cellular level is widely unknown and further research is mandatory.

## Conclusions

ZEK100 and AX30 display degradation characteristics which are, from an engineering point of view, favorable. But the degrading ZEK100 and AX30 implants caused adverse host reactions by inducing an unfavorable osteoclastogenic resorption of bone and a rushed reactive formation of new bone periosteally. Therefore the biocompatibility of ZEK100 and AX30 is questionable and has to be further critically examined. No pronounced influence of the rare earths on the in vivo biocompatibility could be found. A closer assessment of the possible interactions of released degradation products of magnesium alloys has to be done. This should include the interactions on cellular level, especially those with the bone metabolism and the immune system.

## Competing interests

The authors declare that they have no competing interests.

## Authors' contributions

TAH participated in the animal experiment, the μ-computed tomographies and the histological examinations, analysed the data and wrote the manuscript. JR participated in the animal experiment and the design of the study. BvR participated in the histological examinations. DD participated in the animal experiment and helped to draft the manuscript. JMS developed, fabricated and provided the implants. DB developed, fabricated and provided the implants. HW initiated and conceived of the study, participated in its design and coordination. AML initiated and conceived of the study, participated in its design and coordination and participated in the animal experiment. All authors read and approved the final manuscript.
